# Linac-based stereotactic radiosurgery for brain arteriovenous malformations

**DOI:** 10.1186/s13014-022-02130-2

**Published:** 2022-09-29

**Authors:** Ahmed Gawish, Burkard Röllich, Hans-Joachim Ochel, Thomas B. Brunner

**Affiliations:** 1 Department of Radiation Oncology, University Hospital Magdeburg, Leipziger Str. 44, 39120 Magdeburg, Germany; 2 Department of Radiation Oncology, Medical University of Graz, 8036 Graz, Austria

**Keywords:** AVM, SRS, Radiosurgery, Stereotactic radiotherapy, Obliteration

## Abstract

**Purpose:**

Linac stereotactic radiosurgery (SRS) is gaining popularity as a form of radiation treatment for cerebral arteriovenous malformations (AVMs) since the theory of combined radiosurgical and endovascular treatment poses much uncertainty and due to significant technical progress for SRS. This study focuses on how to evaluate obliteration and re-bleeding rates, and to determine factors and adverse effects influencing obliteration after linac-based SRS for cerebral AVMs.

**Material and methods:**

From a statistical record of 71 patients, 31 had partial embolisation, five surgery and 29 had no prior treatment. Using Kaplan–Meier survival and life table analyses, actuarial obliteration and annual bleeding hazard rates were calculated after SRS.

**Results:**

After a follow up of 1, 2 and 3 years the actual obliteration rates were 22, 59 and 66%, respectively whereby it was noted that prior embolization had no effect on the obliteration rate. Annual bleeding hazard rates were further analyzed after stereotactic radiosurgery to be 2.1% and 1.4% for the first and second year respectively. Asymptomatic abnormalities were detected after imaging in 33.9% of patients. A dose of less than 18 Gy significantly reduced the obliteration probability.

**Conclusion:**

SRS is a therapeutic option for intracerebral AVM. In general, there is a low rate of morbidity and a high probability of nidus obliteration.

## Introduction

Arteriovenous malformations (AVM) are congenital collections of irregular, improperly formed cerebral blood vessels that shunt arterial blood directly to the venous system under high pressure, putting patients at often fatal risk of hemorrhage or significant neurological deficit. As an example, the world famous jazz guitarist Pat Martino suffered in 1980 from a near-fatal AVM bleeding leading to near-complete retrograde amnesia of his musical skills which he re-trained over a course of 4 years [1].

The annual bleeding risk from AVMs is estimated to be between 2 and 4%. AVMs in the brain have various chronic effects which include epileptic seizures, neurological dysfunctions and intracranial hemorrhage which is calculated using the Brown formula (risk (%) 105–age) [2].

There are many management choices, including observation, embolized and excised, for these congenital defects. Micro neurosurgery which involves total removal lesions is an efficacious treatment to eliminate AVMs and significantly minimizes the bleeding risk especially in young patients. Stereotactic radiation (SRS) was developed as a treatment tool in the 1970s [3], a method intended to achieve high doses of radiation with steep dose gradients and exact location. Since then, the AVM nest has been developed as a minimally invasive procedure, the goal of which is to eliminate the risk of potential hemorrhage and an appropriate profile of side effects [4].

The majority of the published literature on AVM treated by SRS are series using Gamma Knife device (Elekta) "AB, Stockholm, Sweden). Despite its wide availability, the experiences of Linear Accelerator (LINAC) SRS for AVM are underrepresented. Over time, LINAC-based SRS was technically refined such that this technique is now used more commonly [5].

According to Niccolato [6], 80–90% of obliteration occur in selected cases of lesions smaller than 3 cm. The group of Zabel-du-Bois [7] states that outcomes of stereotactic irradiation of AVMs which are larger are less successful and normally range between 43 and 70%. There is need for more data to add evidence for reliable indications of embolization before radiosurgery since available reports have data that is conflicting between beneficial and deleterious effects of endovascular treatment. This necessitates the need for additional data to confirm the effects of the efficacy and the treatment limits of SRS for this vascular condition in the brain.

This research aimed to analyze our results for cerebral AVM from LINAC-based SRS and to discuss it in the context of the related literature."

## Methods and materials

### Patients

Patients who had SRS for AVM and who had a minimum of 6 months of follow-up were found in a database starting in January 2007. The neurovascular MDT discussed all of the patients (Multidisciplinary team). Patients who were not candidates for microsurgery or embolization were considered for SRS and addressed in the SRS MDT. All post-SRS imaging was reviewed, including Digital Subtraction Angiography (DSA) and Magnetic Resonance Imaging (MRI). A neuroradiologist and a radiation oncologist worked together on this. On DSA, full obliteration was defined as the complete disappearance of the AVM nidus and the feeding channels (defined as gold standard). CTCAE Version 4 was used to examine all case notes for toxicity and to record occurrences.

The radiosurgery-based grading system suggested by Pollock and Flickinger [8] was also employed, and the following formula was applied to determine it: AVM score = 0.1 * AVM volume + 0.02 * age + 0.3 * AVM location. The parameter AVM location’ has a value of 0–2 according to the site of the lesion. The recent simplification of the formula has no discernible effect on the capacity for obliteration prediction. As a result, the standard formula was employed to allow comparisons of our findings with those of previous studies [9].

### Irradiation technique

Patients were treated with a Linac accelerator provided with a micro-multileaf collimator with a 5 mm midpoint leaf width at the isocenter (m3, BrainLab AG, Feldkirchen, Germany) with 6 MV photons. In order to reduce positioning inaccuracies, patients were immobilized with thermoplastic masks with mouth bites and non-invasive fixation with head frames. BrainLab™ (Feldkirchen, Germany) software was used to make plans for radiosurgery. T1-weighted post-contrast, computed tomography FLAIR, angio-magnetic resonance images and magnetic resonance were used to plan target definition.

At the isocenter, the dose was specified, and the entire nidus was described as the target volume as well as previously embolized sections. The dose prescription was adapted according to the closeness of the organs at risk and the target volume. In cases where the AVMs involving to organs at risk, such as the optic chiasm, target scope with a 90% isodose. The patients were administered with doses ranging between 16 and 24 Gy in 1–4 Fractions. Intensity modulation (IMRT) was utilized due to the proximity of organs at risk in twelve patients.

### Follow-up

The first clinical examination was carried out four to six weeks after treatment and first control MRI after irradiation was after six months. After every 6 months the patient had follow-up magnetic resonance imaging.

The control examinations were taken yearly when obliteration was diagnosed. Then the imaging examinations after treatment were terminated. Obliteration was assessed through either magnetic resonance or DSA.

### Statistics

Kaplan–Meier estimations were used to assess actuarial obliteration rates while the annual hazard rates of hemorrhage were derived using Kaplan–Meier life table. Statistica 7.1 PL program was used for all calculations and the significant level was set to 0.05. The significance of the selected parameters was determined by the Kaplan–Meier survival analysis according to their type of parameter and to the extent they could affect the obliteration rate. For independent samples Student’s t-test, Chi-square and Mann–Whitney U test were used for comparison.

## Results

### Patients, AVM characteristics and treatment

A total of 71 patients who underwent radiosurgery between January 2007 to December 2018 were identified in our study. We excluded 3 patients because of inadequate follow-up.

Of these, 67 patients suffered from single AVM and just one patient suffered from 2 AVM. The median age at the time of the radiosurgery was 39 years (mean 42, range 7–78 years). Four patients were under 18 years old. Male to female ratio was 1.26:1, with 38 Males and 30 females. Table 1

**Table 1:** Characters of the patients

Variable		Range
Gender	Male: 38	
Male	Female: 30	
Age (years)	Median: 39	7–78
	Mean: 42	
Maximum diameter (mm)	Median: 27.8	9.45–79.90
	Mean: 25.35	
Volume of lesion (cc)	Median: 6 ml	0.6–64.86
	Mean: 10.6 ml	
Time to CO(months)	Median: 19.5	6–60 months
	Mean: 16	
Pre-SRS DAS	45/68	
Pre-SRS operation	12/68	
Pre_SRS haemorrhage	59/68	
Pre-SRS seizures	10/68	
Post-SRS Bleeding	3/68	
Post-SRS seizures	2/68	
Post-SRS re-treatment	2/68	

The pre-radiosurgery symptoms differed between the patients, 58/68 patients were suffered from hemorrhage, 10/68 showed epilepsy, while 5/68 patients reported only headaches.

**Fig. 1:**
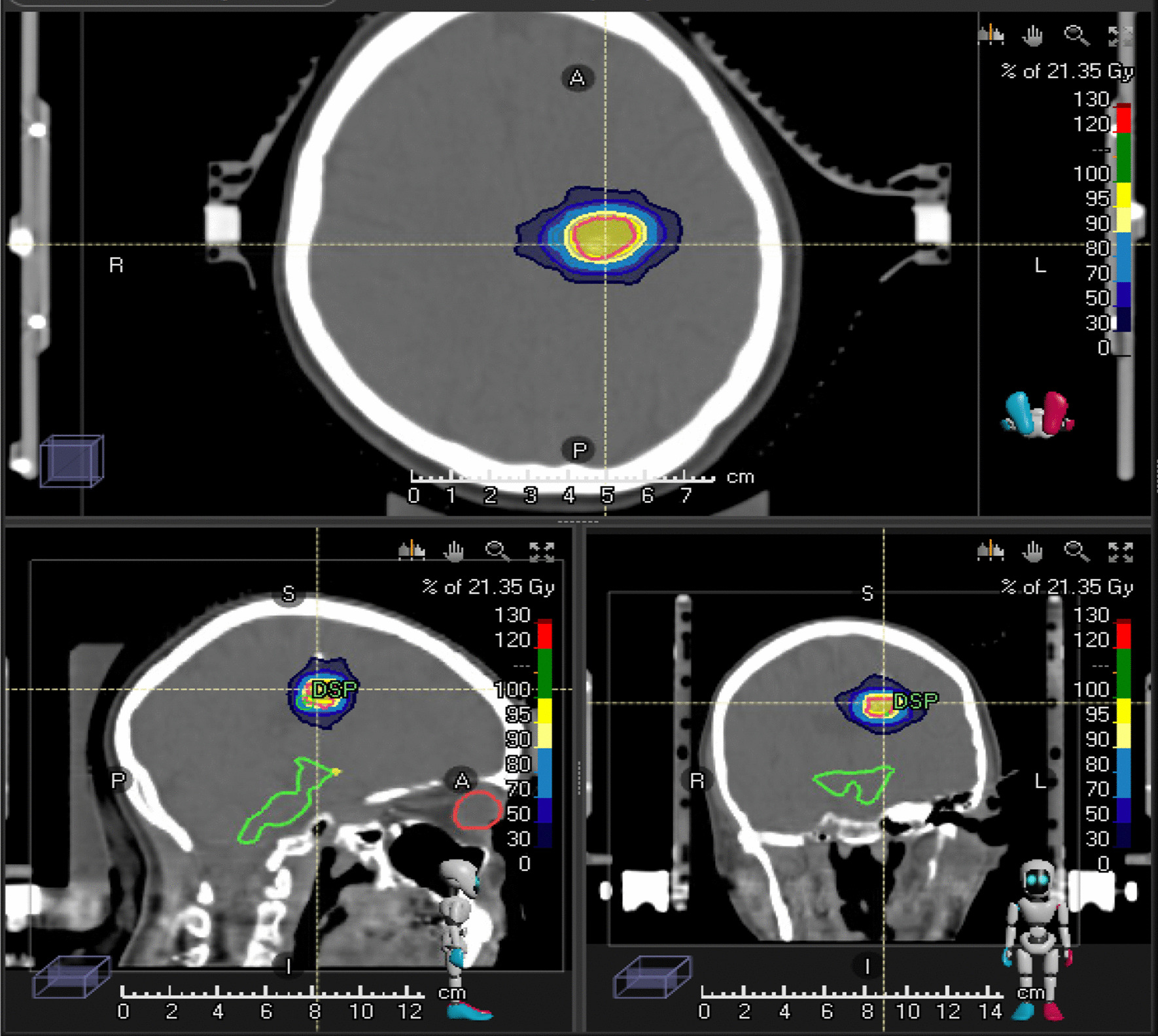
Treatment plan of 20 Gy SRS for AVM

The mean volume was 10.6 cm^3^ (range 0.26–64.86 cm^3^, median 6 cm^3^). The median maximum dimeter 25 mm (range 9–70 mm, mean 27 mm). The median radiosurgery dose was 20 Gy (range 16–24, mean 19.1 Gy). The radiosurgery was performed in a median of a single fraction (range 1–4), 60 patients received 1 fraction, 6 patients received 2 fractions while 2 patients received 4 fractions. A single isocentre technique was used in 64 patients (94%) and the most common prescription isodose line was 90%. The median normal brain volume V12 Gy was 4.5 ml (Fig. 1).

Eleven lesions had an AVM score of one, forty-four had an AVM score between one and two, and eighteen had an AVM score more than two.

### Obliteration

**Fig. 2:**
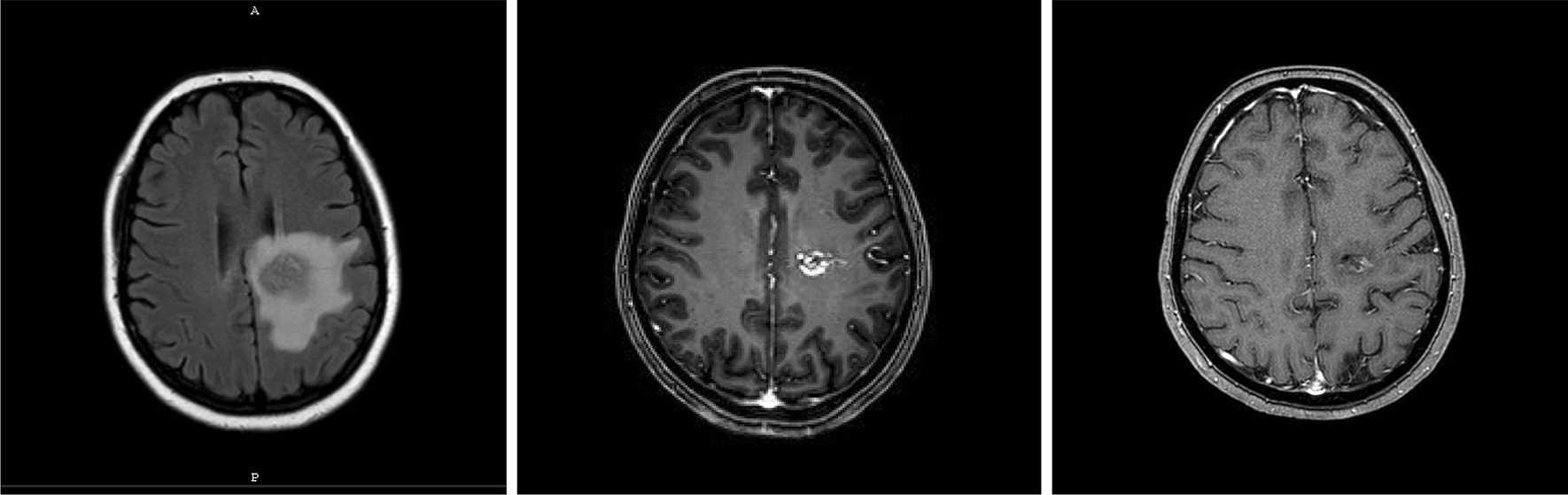
Post-SRS follow up (1) MRI shows post-SRS oedema (2) Partial obliteration (3) complete obliteration

After 1, 2 and 3 years, the obliteration rates were 22, 59 and 66%, sequentially. A mean follow up of 35 months (median 26, range 6–120 months) was performed, (46/68) 68% of the patients showed complete obliteration, 9/68 patients (13%) showed partial obliteration without any bleeding symptoms, while 19% (13 patients showed no obliteration after radiosurgery. 3/68 patients suffered from intracranial hemorrhage after 2 years of SRS. Only one patient needed a surgery (Fig. 2).

**Fig. 3:**
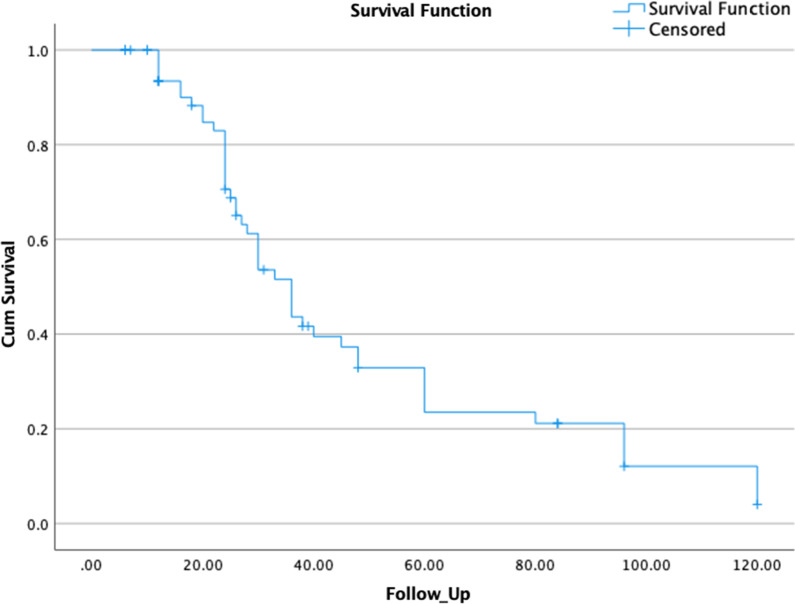
Kaplan–Meier obliteration curve for arteriovenous malformations treated with radiosurgery

The median time to radiological obliteration was 19.5 months, range from (6–60 months) (Fig. 3).

The prior operation, DSA, age, dosage, and maximal diameter of the 68 AVM treated with radiosurgery were examined for associations with time to AVM obliteration (Table 2). The higher the dosage was, the shorter was the duration to obliteration (HR 0.97; *p* 0.048). AVMs with a larger diameter had a longer time to obliteration (HR 0.95; *p* = 0.003). When obliteration was diagnosed through angiography or MRI, only dosage and diameter remained significant predictors of obliteration rates. The history of embolisation did not influence the probability of obliteration, however the history of operation did (Table 2).

**Table 2:** Factors affecting the obliteration

Factor	p value	95% confidence interval
Age (years)	0.205	0.946–1.015
Dose (Gy)	0.048	0.920–1.089
Maximum diameter (mm)	0.003	0.895–0.999
DSA before SRS	0.199	1.052–3.229
Operation before SRS	0.009	0.320–1.089

### Treatment complications

There was no treatment-related mortality. Radiation caused new neurological issues (hemiparesis, sensory deficiency, and memory impairment) were not reported. Transitory effects included temporary baldness as a result of radiation and scalp numbness, which was most likely caused by the stereotactic pins.

Two patients experienced seizures shortly after SRS which were treated with antiepileptic medication.

Following therapy, 3/68 patient developed bleeding. During the follow up (at 3 years), two patients experienced a bleeding that did not necessitate surgical removal. The bleeding in the third patient happened 4 years after radiosurgery, and the surgical removal was the option of choice. Due to the low frequency of bleeding during stereotactic radiosurgery, no valid statistical study aimed at identifying risk factors for bleeding could be conducted.

Asymptomatic imaging abnormalities were found in 39% of the patients. They were defined as T1-hypointensity, T2-hyperintensity or regions of contrast enhancement. Radionecrosis was discovered in 14/68 patients (20%). There was no correlation between the incidence of radio-necrosis and the irradiated volume or previous treatment (*p* = 0.21, *p* = 0.70).

## Discussion

This retrospective clinical series confirms the therapeutic flexibility, clinical effectiveness and low toxicity of LINAC-SRS, regardless of a plethora of parameters: Patient age, surgical accessibility, AVM position, prior care and, to lesser degree, size for cerebral AVM treatment in the neurosurgical armamentarium.

The reported CO rates following SRS in the literature vary significantly, according mostly to the single dosage used and the size of the AVM [10, 11]. Flickinger et al. [12] found CO in 73% of 264 AVM following gamma knife SRS and developed a dose–response curve for AVM obliteration with a maximal overall obliteration rate of 88% and limited improvement above 25 Gy. Friedman et al. [13] published a study on 158 individuals with cerebral AVM who were treated with linac-based SRS. CO was found in 81% of AVM ranging from 1 to 4 cc in volume, 89% of AVM ranging from 4 to 10 cc in volume, and 69% of AVM bigger than 10 cc in volume.

In a single institution, we treated 68 patients with cerebral AVM using linac-based SRS. After 2 years, the actuarial CO rate was 59%, and after 3 years, it was 66%. The median time to CO was 19.6 months (range 6–60 months). The rate of CO; are slightly lower than is commonly reported in the literature, and this can be attributable to the comparatively large mean volume of treated lesions. However, they are comparable to the obliteration rates reported by Zabel-du Bois et al. [14] In a cohort of 65 patients, they found a 50% actuarial 3-year obliteration rate with a median time to obliteration of 22.4 months.

Furthermore, the mean volume of the nidus in their series was 5.2 cm^3^, which is about half the size of as the volume described in our study (10.6 cm^3^). The same group of researchers also reported obliteration rates of big AVMs treated with radiosurgery or hypofractionated radiation [15]. The actuarial 3- and 4-year obliteration rates following radiosurgery were 47 and 60%, respectively [15]. The median volume in our series was 6 cm^3^, whereas the median target volume in the literature was 7.1 cm^3^. It should be emphasized that the mean and median doses reported in our study were 19 Gy and 20 Gy, whereas the median dosage in their series was 17 Gy with a range of 15–19 Gy administered on the 80% (surrounding) isodose. The inferior treatment outcomes in this report compared to us may be attributed to the use of lower dosages, particularly on the nidus’s periphery, since we defined the dose at the isocenter.

In general, cerebral AVM is associated with an annual bleeding risk of 2–4% [16]. The risk of recurring bleeding has been observed to be greater (6%) in the first year following first hemorrhage and to rapidly reduce thereafter [17]. In the research by Sasaki et al., the yearly rebleeding rate was 13% [18]. The morbidity and death rates in rebleeding patients were 12.5% and 62.5%, respectively. Because the prognosis in untreated patients with severe AVM and a history of bleeding is poor, the authors propose that patients who have a hemorrhage be treated to prevent a second bleeding. SRS appears to be an effective method of reducing bleeding risk in deep AVM till full obliteration [15, 19].

Zabel-du Bois and colleagues [14] observed that the yearly bleeding risk following radiosurgery was 4.7, 3.4, and 2.7% over the first, second, and third years of surveillance, respectively. These numbers are consistent with our findings and support the concept that the risk of bleeding reduces progressively following SRS. Zabel-du Bois et al. [20] recently validated the trend of an annual bleeding hazard to decrease following stereotactic radiosurgery in an investigation of a group of patients irradiated after partial embolisation of the nidus. The yearly bleeding risk in that group was 4.4, 2.2% after first and 2 years and declined to 1.7% during the third year of follow-up [19].

In our series, only three patients (4.4%) had intracranial haemorrhage after radiosurgery, 18, 23 and 28 months after treatment. In spite of bleeding, the neurological status of the patients did not change significantly, and no new neurological deficits were diagnosed. All of them had bled before radiosurgery and had partial embolisations before radiosurgery. The patients were irradiated with 20 Gy. Only three patients in our series had bleeding following stereotactic radiosurgery. The yearly bleeding hazard rate of 2.1% and 1.4% in the first and second years of observation, respectively, is comparable to what has been reported in the literature.

In our study, 59% of obliterations were identified after 2 years of follow-up and 66% after 3 years, indicating that the latency period may be greater than the usually reported 2–3 years. This is especially true for big AVMs. This is supported by other investigations, which indicate that the latency period can persist up to 4–5 years [7, 15, 21, 22]. Touboul et al. [21] discovered that in a sample of 100 patients, obliteration was identified in 40% of patients after 3 years of observation, but it was 62% after 5 years of follow-up, a 50% increase with 2 more years of follow-up [21]. Zabel-du Bois et al. also validated these findings. In their series, the obliteration rate was 47% after 3 years of monitoring, but it increased to 60% after 4 years [15].

The investigation of the effect of prior embolisation on obliteration rates revealed that irrespective of previous endovascular intervention, irradiation outcomes are comparable. It contradicts several research claiming that prior embolisation lessens the likelihood of obliteration. It can be explained by the fact that we designated the goal volume as the entire nidus, whereas most other studies defined the target volume based on the angiographic look of the post-embolisation nidus. This may result in underdosing of previously embolised AVM parts and, as a result, an increased risk of re-vascularisation [23, 24]. Recent data indicate that earlier embolisation lowers the obliteration rate if the embolised region of the nidus is excluded from the target volume [25], which supports our approach of treating the whole AVM nidus. We believe that irradiating the whole nidus counterbalanced the proangiogenic impact of embolisation, resulting in the identical obliteration rates in patients with embolised and non-embolised AVMs in our study.

The implementation of a radiosurgery-based AVM score is also supported by current research. Patients with a low AVM score had the best outcomes, but an AVM score of 2 or above indicates a poor treatment outcome. Pollock and Flickinger [9] estimate that an AVM score of 2 has a 50% risk of obliteration. According to our data, the obliteration rate in the group with an AVM score less than 2 was 65% (33/51). The obliteration rate reduced to 33% (6/18) if the AVM score was > 2. Similarly, in a sample of 56 patients treated by Pollock et al. [26] for AVMs in the basal ganglia, thalamus, or brainstem with a median AVM score of 1.83, 43% of lesions were cured following a single treatment and 57% after multiple surgeries. The greatest outcomes were found in the group with an AVM score of 1.5, with a 67% obliteration rate [26]. The AVM score has also been found to be beneficial in predicting the risk of cerebral hemorrhage following radiosurgery [14]. The incidence of hemorrhage following radiosurgery was low in our dataset, making statistical analysis difficult. As a result, we were unable to demonstrate the impact of AVM score on bleeding risk.

Blamek and colleagues [27] showed, using Kaplan–Meier analysis, that irradiation with a single dose less than 15 Gy results in a considerable decrease in the actuarial obliteration rate when compared to larger doses [27]. According to Nataf et al. [28], irradiation with doses of 15 Gy resulted in a 44% obliteration rate, whereas irradiation with doses of 15–20 Gy resulted in an 89% obliteration rate. The SRS was well tolerated, and no persistent neurological impairments were found. Two individuals experienced transient neurological issues that were caused by the initial effects of irradiation and disappeared after two or three weeks. Imaging abnormalities (edema or necrosis) were seen in 33.9% of instances after stereotactic radiosurgery [29]. This result is comparable with previous studies that estimate the incidence of imaging abnormalities at one-third to one-half of irradiated individuals [30, 31].

At the time of SRS, 5.8% (4/68) of the patients in this research were between the ages of 7 and 18. On angiography, 75% (3/4) of the patients had obliteration. There was no evidence of bleeding. Pediatric AVM obliteration rate has been linked to marginal dose and AVM volume [32, 33], much like in adults. There were too few patients in our research to reliably analyze outcomes related to the pediatric population. However, Kano et al. [32] discovered a 5-year rate of full angiographic obliteration of 67% with a 1.8% yearly bleeding risk in the latent period in a study of 135 pediatric patients. Shin et al. [33] discovered that AVM in the cerebellum and feeding arteries in the posterior fossa increased the incidence of latent period hemorrhage. Given the parallels to adult AVM, there is support for identical treatment standards for adults, but with longer follow-up due to a pediatric population's larger total life expectancy [7]. The comprehensive analysis of clinical outcomes and CO as shown in Table 3 show that despite the obvious difference in dose description institutes, we cannot establish that one platform provides a superior clinical outcome.

**Table 3:** Recent studies of Linac based SRS for AVM

Study	No	Obliteration rate (%)	Dose (median)	Treatment volume	SRS system	Reported toxicity (%)	Follow up (median) years
Bollet et al. [36]	118	54	24.5 Gy	7.4 cc	Linac	3.9	3.8
Zebel Du Bois et al. [10]	50	76	18 Gy	4 cc	Linac	12	3.1
Gobin et al. [24]	125	65	25 Gy	6.2 cc	Linac	3	3.3
Miyawaki et al. [5]	73	50	25.18 Gy/max	8.4 cc	Linac	22	5.9
Yahya et al. [37]	47	74.5	19.8 Gy	1.97 cc	Linac	6.4	4.4
Schlienger et al. [23]	169	64	25 Gy	2.46 cc	Linac	2.3	4.8
Current	68	68	19.8 Gy	10.6 cc	Linac	20	2.1

There was a large heterogeneity in the group and radiation doses were moderate with a mean dose of 19 Gy, which may be considered low to ensure an optimal treatment effect. Dose reductions are a consequence of large AVM volumes providing the risk of adverse effects that are associated with the application of higher doses to large volumes of the brain. According to Laakso et al. [34] even partial treatment reduces mortality in patients with AVMs and may be suitable for surgery. Neoadjuvant irradiation can facilitate the surgical procedure and reduce morbidity. Sanchez-Mejia et al. [35] stated that follow ups should be longer for a more reliable assessment on treatment on bleeding risk, long-term obliteration rate and AVM-associated mortality.

## Conclusions

This study adds to the body of knowledge about the usefulness of SRS as a therapeutic option for intracerebral AVM. In general, there is a low rate of morbidity and a high probability of nidus obliteration. The treatment dosage and the nidus's greatest diameter were the biggest drivers of obliteration. Additional consideration should be given to bigger AVMs, since they have a poorer obliteration rate regardless of whether they are treated with radiosurgery or fractionated radiotherapy.

## Data Availability

The datasets used and analyzed during the current study are available from the corresponding author on reasonable request.

## Abbreviations

AVM: Arterio-vascular malformation
SRS: Radiosurgery
DSA: Digital subtraction angiography
MRA: Magnetic resonance angiogram
MRI: Magnetic resonance imaging
MDT: Multidisciplinary team
CO: Complete obliteration
